# Oxidative stress and cardiometabolic biomarkers in patients with non-alcoholic fatty liver disease

**DOI:** 10.1038/s41598-021-97686-6

**Published:** 2021-09-16

**Authors:** Aleksandra Klisic, Nebojsa Kavaric, Ana Ninic, Jelena Kotur-Stevuljevic

**Affiliations:** 1 Center for Laboratory Diagnostics, Primary Health Care Center, University of Montenegro-Faculty of Medicine, Trg Nikole Kovacevica 6, 81000 Podgorica, Montenegro; 2 Department for Medical Biochemistry, University of Belgrade-Faculty of Pharmacy, Belgrade, Serbia

**Keywords:** Biomarkers, Gastroenterology

## Abstract

Oxidative stress is assumed to be the underlying feature of non-alcoholic fatty liver disease (NAFLD). To our knowledge, the mutual involvement of redox status homeostasis parameters [i.e., advanced oxidation protein products (AOPP), pro-oxidant-antioxidant balance (PAB), total oxidant status (TOS), total antioxidant status (TAS) and oxidative-stress index (OSI)] and cardiometabolic biomarkers in subjects with NAFLD has not been examined yet. Accordingly, we aimed to investigate this potential relationship. A total of 122 subjects with NAFLD were compared with 56 participants without NAFLD. The diagnosis of NAFLD was confirmed by abdominal ultrasound. Anthropometric and biochemical parameters were measured. OSI, Castelli’s Risk Index I (CRI-I) and Castelli’s Risk Index II (CRI-II) were calculated. Univariate and multivariate binary logistic regression analysis were used to test the predictions of oxidative stress and cardiometabolic markers, respectively for NAFLD. Principal component analysis (PCA) was applied to explore its mutual effect on NAFLD status. Significant positive associations of CRI-I, CRI-II, high sensitivity C-reactive protein (hsCRP) and AOPP with NAFLD were found. PCA analysis extracted 3 significant factors: Oxidative stress-cardiometabolic related factor (i.e., triglycerides, AOPP, HDL-c and HbA1c)-explained 36% of variance; Pro-oxidants related factor (i.e., TOS and PAB)-explained 17% of variance; and Antioxidants related factor (i.e., TAS)-explained 15% of variance of the tested parameters. Moreover, binary logistic regression analysis revealed significant predictive ability of Oxidative stress-cardiometabolic related factor (*p* < 0.001) and Pro-oxidants related factor (*p* < 0.05) for NAFLD status. In addition to oxidative stress (i.e., determined by higher AOPP levels), dyslipidemia (i.e., determined by higher lipid indexes: CRI-I and CRI-II) and inflammation (determined by higher hsCRP) are independently related to NAFLD status. The mutual involvement of pro-oxidants (i.e., TOS and PAB), or the joint involvement of pro-oxidants (i.e., AOPP) and cardiometabolic parameters (i.e., HbA1c, triglycerides and HDL-c) can differentiate subjects with NAFLD from those individuals without this metabolic disorder. New studies are needed to validate our results in order to find the best therapeutic approach for NAFLD.

## Introduction

It is well known that non-alcoholic fatty liver disease (NAFLD) represents the most common form of liver disorders. Although not all subjects with NAFLD develop non-alcoholic steatohepatitis, it is important to note that many diseases are related to this metabolic disorder, such as liver fibrosis, cirrhosis and hepatocellular carcinoma, but also diabetes mellitus type 2 (T2DM) and cardiovascular disease^1,2^.

The prevalence of NAFLD is increasing rapidly worldwide, in parallel with the increase in the prevalence of obesity, sedentary lifestyle and increase in energy intake^2,3^.

Oxidative stress is presumed to be the major initiator of activation of inflammatory cascade derived from increased visceral adipose tisue^3–5^. Adipokines and cytokines make an impact on insulin signalling pathways leading to overproduction of reactive oxygen/nytrogen species (ROS/RNS) and consequent impaired insulin sensitivity. All these processes, although previously investigated by many researches give different conclusions, since no universial redox homeostasis parameter to diagnose NAFLD has been described, nor the best therapeutic approach has been established yet^1–5^.

Insulin resistance, a major determinant of metabolic processes in NAFLD, favors the release of free fatty acids (FFA) from visceral adipose tissue into the liver. Increased FFA flux favours dyslipidemia and triggers peroxidation of lipids. Atherogenic dyslipidemia, characterized by high levels of triglycerides (TG), low levels of high density lipoprotein cholesterol (HDL-c), high levels of low density lipoprotein cholesterol (LDL-c), and high number of circulating small-dense LDL (sdLDL) particles, is the common finding in NAFLD patients^6^. The mentioned form of dyslipidemia may lead to enhanced activity and expression of sterol regulatory element binding protein-1c (SREBP1-c) that modulates the gene expression related to adipocytes differentiation, FFA oxidation and lipogenesis, which further contribute to unfavorable lipid profile in NAFLD^6^.

To our knowledge, the mutual involvement of redox status parameters in addition to cardiometabolic biomarkers in subjects with NAFLD has not been examined yet.

Therefore, the deeper recognition of metabolic processes related to NAFLD would enable us to diagnose it and treat it adequately, much before its complications occur.

We hypothesize that its joint measurement may provide better insight into the timely diagnosis of NAFLD. It is especially important, since many individuals exhibit normal values of liver enzymes (i.e., transaminases) and no clinical symptoms at the moment when NAFLD occurs^1^. That is the reason why NAFLD is often unrecognized. Hence, in an attempt to give our contribution to further elucidation of pathophysiological mechanisms of NAFLD, we aimed to examine the mutual relationship between redox status homeostasis and cardiometabolic parameteres in individuals with NAFLD.

## Patients and methods

### Subjects

A total of 122 subjects with NAFLD were compared with 56 age-matched participants without NAFLD. The participants were included consecutively. The research was conducted in a period between May–October 2019, after obtaining the approval of the Ethics Committee of the Primary Health Care Center, Podgorica, Montenegro and after providing the written informed consent of the examinees. All the participants filled in the questionnaire about lifestyle habits, demographic data and acute/chronic diseases. Each participant underwent anthropometric [i.e., body weight and height, waist circumference (WC), and body mass index (BMI)] and blood pressure measurement [i.e., systolic (SBP) and diastolic blood pressure (DBP)].

The diagnosis of NAFLD was confirmed by abdominal ultrasound performed by experienced radiologist^7^.

The inclusion criterion for the case group was the presence of NAFLD diagnosed by abdominal ultrasound. The exclusion criterion for the case group was the absence of NAFLD confirmed by abdominal ultrasound. The exclusion criteria for all participants were: subjects younger than 18 years, endocrine disorders other than diabetes, acute infection, high sensitivity C-reactive protein (hsCRP) higher than 10 mg/L, liver disorders other than steatosis, malignant diseases, severe anaemia, ethanol consumption > 20 g/day, use of glucocorticoids, non-steroidal anti-inflammatory medications and/or antibiotics, pregnant women and unwillingness to participate in the research.

### Methods

The blood samples were taken in the morning after a fast of at least 8 h. One blood sample was obtained in the tube with K_2_EDTA for measurement of glycated haemoglobin (HbA1c) levels and the other was collected in the tube with serum separator and clot activator for the measurement of oxidative stress and biochemical parameters. After being left to clot for 30 min, sera samples were obtained by centrifugation at 2000 g for 10 min.

Biochemical parameters [i.e., total cholesterol (TC), HDL-c, LDL-c, TG, fasting glucose, HbA1c, hsCRP, aspartate aminotransferase (AST), alanine aminotransferase (ALT), gamma glutamyl transferase (GGT)] were measured immediately on Roche Cobas c501 chemistry analyzer (Roche Diagnostics GmbH, Mannheim, Germany) by standard procedures, whereas one aliquot of sera was frozen and kept at -80 °C until analysis for oxidative stress parameters was conducted. Castelli’s Risk Index I (CRI-I) and Castelli’s Risk Index II (CRI-II) were calculated as follows: CRI- I = TC/HDL-c and CRI-II = LDL-c/HDL-c, respectively^8^.

Parameters of oxidative stress status were determined as follows:

A reaction with potassium iodide and glacial acetic acid was applied for advanced oxidation protein products (AOPP) measurement by the method of Witko-Sarsat et al.^9^. A 3,3’, 5,5’-tetramethylbenzidine as a chromo gen by the method of Alamdari et al. was used for pro-oxidant-antioxidant balance (PAB) levels measurement^10^. Total oxidant status (TOS) was determined with o-dianisidine, whereas ABTS as a chromogen was used for the measurement of total antioxidant status (TAS)^11,12^. Oxidative-stress index (OSI) was obtained as described previously: OSI (arbitrary unit) = TOS (μmol H_2_O_2_ equivalent/L)/TAS (μmol Trolox equivalent /L) × 100^13^.

### Statistical analysis

Statistical analyses were conducted using SPSS version 21.0 (SPSS Inc., Chicago, USA). Distribution of continuous variables were tested by Shapiro Wilk and Kolmogorov Smirnov tests and the differences between them were assessed using Student *t*-test and Mann–Whitney *U*-test.

The data were expressed as mean ± standard deviation (SD) and median (interquartile range). Categorical data were tested using Chi-square test for contingency tables and presented as absolute frequencies. Associations between oxidative stress and cardiometabolic markers (independent, continuous variables) and NAFLD (categorical dichotomous variables: 0 – no NAFLD and 1 – NAFLD) were examined using univariate and multivariate binary logistic regression analysis. Data were presented as Odds Ratios (OR) and 95% Confidence Intervals (CI).

Principal component analysis (PCA) with varimax rotation was employed to reduce the number of variables to adequate number of factors similar by the level of variation. Factor extraction was determined for Eigenvalue larger than 1. Criterion for variables inclusion in distinct factor was factor loadings larger than 0.5. PCA analysis also enables us to calculate scores for factors, in order to use those scores in subsequent binary logistic regression analysis for testing statistical significance of NAFLD predictors.

Statistical significance was set at two-tailed P level less than 0.05.

### Ethical statement

This is to confirm that all methods were carried out in accordance with relevant guidelines and regulations.

## Results

Clinical characteristics of the population stratified by the presence of NAFLD are displayed in Tables 1 and 2.

**Table 1:** General data of participants according to NAFLD status.

	Non-NAFLD N = 56	NAFLD N = 122	*p*
N (male/female)	5/51	58/64	< 0.001
Age, years^#^	60.0 ± 10.3	62.7 ± 9.7	0.064
BMI, kg/m^2^	24.0 (23.1–25.3)	31.5 (29.9–34.1)	< 0.001
WC, cm	85 (81–88)	105 (101–112)	< 0.001
SBP, mmHg	136 (126–151)	131 (126–145)	0.410
DBP, mmHg	85 (77–93)	84 (78–92)	0.710
Glycemic status, (normoglycemic/prediabetes/diabetes)	41/7/8	40/24/58	< 0.001
Smoking status (no/yes)	44/12	101/21	0.502
Antihyperglycemics, (no/yes)	50/12	72/50	< 0.001
Insulin therapy, (no/yes)	50/1	106/16	0.017
Antihypertensives, (no/yes)	33/23	32/90	< 0.001
Hypolipemics, (no/yes)	42/14	75/47	0.077

Data are presented as median (interquartile range) and compared with Mann–Whitney *U*-test.

^#^Normally distributed data are presented as arithmetic mean ± standard deviation and compared with Student *t*-test.

Categorical variables are presented as absolute frequencies and compared by Chi-square test for contingency tables.

BMI-Body mass index; WC-Waist circumference; SBP-Systolic blood pressure; DBP-Diastolic blood pressure.

**Table 2:** Biochemical and oxidative stress status markers of examined population according to NAFLD status.

	Non-NAFLD N = 56	NAFLD N = 122	*p*
Glucose, mmol/L	5.5 (5.2–5.8)	6.4 (5.5–8.3)	< 0.001
HbA1c, %	5.3 (5.1–5.6)	6.0 (5.5–7.0)	< 0.001
TC, mmol/L	5.93 (4.86–6.66)	5.92 (4.93–6.88)	0.612
HDL-c, mmol/L^#^	1.78 ± 0.41	1.21 ± 0.30	< 0.001
LDL-c, mmol/L	3.50 (2.71–4.07)	3.43 (2.76–4.34)	0.383
TG, mmol/L	1.15 (0.94–1.39)	2.28 (1.73–2.95)	< 0.001
CRI-I	3.30 (2.77–3.68)	4.98 (4.15–5.96)	< 0.001
CRI-II	1.98 (1.50–2.33)	2.93 (2.41–3.79)	< 0.001
hsCRP, mg/L	0.52 (0.32–1.07)	1.73 (0.79–3.11)	< 0.001
AST, U/L	20 (17–22)	19 (17–24)	0.838
ALT, U/L	15 (11–20)	23 (17–31)	< 0.001
GGT, U/L	11 (9–15)	23 (16–31)	< 0.001
AOPP, μmol/L	32.10 (30.60–38.65)	49.35 (40.00–62.00)	< 0.001
PAB, HKU	119 (97.97–142)	102 (66.87–129)	0.068
TOS, μmol/L H_2_O_2_ equivalent/L	6.30 (4.10–18.33)	12.70 (7.50–19.20)	0.003
TAS, μmol/L Trolox equivalent/L	1170 (1075–1269)	1212 (1136–1318)	0.042
OSI, arbitrary unit	0.51 (0.35–1.54)	1.06 (0.58–1.65)	0.008

Data are presented as median (interquartile range) and compared with Mann–Whitney *U*-test.

^#^ Normally distributed data are presented as arithmetic mean ± standard deviation and compared with Student *t*-test.

Categorical variables are presented as relative frequencies and compared by Chi-square test for contingency tables.

HbA1c-Glycated hemoglobin; TC-Total cholesterol; HDL-c-High density lipoprotein cholesterol; LDL-c-Low density lipoprotein cholesterol; TG-Triglycerides; CRI-I-Castelli’s Risk Index I; CRI-II-Castelli’s Risk Index II; hsCRP-High sensitivity C–reactive protein; AST-Aspartate aminotransferase; ALT-Alanine aminotransferase; GGT-Gamma glutamyl transferase; AOPP-Advanced oxidation protein products; PAB-Prooxidant-antioxidant balance; TOS-Total oxidant status; TAS-Total antioxidant status; OSI-Oxidative-stress index.

Study participants were of a similar age. Significantly more female than male participants were among those with NAFLD. Also, more subjects with prediabetes and diabetes, users of antihyperglycemic, insulin and antihypertensive therapies were in NAFLD than in non-NAFLD group. Compared to non-NAFLD group, participants with NAFLD had higher anthropometric indexes (BMI and WC) (Table 1).

Levels of biochemical markers such as glucose, HbA1c, TG and hsCRP were higher, but HDL-c levels were lower in patients with NAFLD. As expected, the activities of liver enzymes (GGT and ALT) were higher in NAFLD patients than in those without NAFLD. The same was obvious for the calculated lipid indexes, CRI-I and CRI-II. Oxidative stress was evident in NAFLD group. It was demonstrated by higher AOPP, TOS and OSI levels. However, as antioxidative stress marker, TAS was also higher in NAFLD group of patients (Table 2).

Our further intention was to investigate whether calculated lipid indexes, inflammatory and oxidative stress markers were associated with NAFLD presence (Table 3). Univariate binary regression analysis revealed significant predictive capability of CRI-I, CRI-II, hsCRP, AOPP and TOS for NAFLD status, demonstrated by the following OR, respectively 4.063, 3.548, 1.835, 1.115 and 1.057. Of all, CRI-I and AOPP as independent factors were able to explain the highest variation of 44.7% and 43.8% in NAFLD presence, respectively.

**Table 3:** Estimated odds ratios after binary logistic regression analysis for NAFLD groups as dependent variable.

Single predictors	OR (95% CI)	*p*	Nagelkerke R^2^
**Unadjusted**			
CRI-I	4.063 (2.554–6.463)	< 0.001	0.447
CRI-II	3.548 (2.184–5.771)	< 0.001	0.296
hsCRP, mg/L	1.835 (1.328–2.537)	< 0.001	0.174
AOPP, μmol/L	1.115 (1.098–1.216)	< 0.001	0.438
PAB, HKU	0.993 (0.985 – 1.002)	0.124	0.019
TOS, μmol/L	1.057 (1.013–1.103)	0.011	0.068
TAS, μmol/L	1.001 (1.000–1.003)	0.099	0.022
OSI, arbitrary unit	1.128 (0.869–1.465)	0.365	0.008

Predictors in Models	OR (95% CI)	*p*	Nagelkerke R^2^
**Adjusted**			
CRI-I	3.614 (2.224–5.873)	< 0.001	0.600
CRI-II	3.351 (1.985–5.659)	< 0.001	0.528
hsCRP, mg/L	1.706 (1.207–2.411)	0.002	0.438
AOPP, μmol/L	1.145 (1.084–1.213)	< 0.001	0.570
TOS, μmol/L H_2_O_2_ equivalent/L	1.027 (0.988–1.068)	0.179	0.375

Data are given as OR (95% CI).

Models included each marker and categorical variables (glycemic status, antihypertensive therapy and gender).

CRI-I-Castelli’s Risk Index I; CRI-II-Castelli’s Risk Index II; hsCRP-High sensitivity C–reactive protein; AOPP-Advanced oxidation protein products; PAB-Prooxidant-antioxidant balance; TOS-Total oxidant status; TAS-Total antioxidant status; OSI-Oxidative-stress index.

All tested markers which had significant OR in univariate analysis were tested in multivariate analysis together with categorical data that were significantly different between tested groups as covariables (glycemic status, antihypertensive therapy and gender). CRI-I, CRI-II, hsCRP and AOPP kept independent significant predictive capability for NAFLD status, while TOS lost it.

PCA analysis was implemented on redox status parameters, lipid status parameters and HbA1c to find a relation between oxidative stress, dyslipidemia, insulin resistance and NAFLD. PCA model appropriateness was confirmed with Keiser-Meier-Olkin measure of sample adequacy (KMO index = 0.697) and Bartlett’s test of sphericity is significant (*p* < 0.001). After initial analysis total cholesterol was excluded because of low individual measure of sampling adequacy (anti-image correlation coefficient < 0.5). The final model was presented in the Table 4.

**Table 4:** Factors extracted by principal component analysis with percent of variability and variables’ loadings.

Factors	Variables (loadings)	Factor variability
Oxidative stress-cardiometabolic related factor	TG (0.850) AOPP (0.839) HDL-c (–0.731) HbA1c (0.621)	36%
Pro-oxidants related factor	TOS (–0.781) PAB (0.719)	17%
Antioxidants related factor	TAS (–0.859)	15%

TG-Triglycerides; AOPP-Advanced oxidation protein products; HDL-c-High density lipoprotein cholesterol; HbA1c-Glycated hemoglobin; TOS-Total oxidant status; PAB-Pro-oxidant-antioxidant balance; TAS-Total antioxidant status.

This analysis extracted 3 significant factors with total percent of explainable variation of 68% of the investigated parameters. The first factor (i.e. Oxidative stress-cardiometabolic related factor) explained 36% of variance. The second factor (i.e. Pro-oxidants related factor) explained 17% of variance, and the third factor (i.e. Antioxidants related factor) explained 15% of variance. By using scores derived from PCA we performed binary logistic regression analysis and revealed significant predictive capability of Oxidative stress-cardiometabolic related factor (i.e. TG, AOPP, HDL-c and HbA1c, *p* < 0.001) and Pro-oxidants related factor (TOS and PAB, *p* < 0.05) towards NAFLD status. Results of univariate binary logistic regression analysis are presented at the Table 5.

**Table 5:** Binary logistic regression analysis of predictors of NAFLD status.

Predictors	B (SE)	Wald	OR (95% CI)	*p*
Oxidative stress-cardiometabolic related factor	4.25 (0.732)	33.8	70.5 (16.8–295.9)	< 0.001
Pro-oxidants related factor	-0.454 (0.230)	3.9	0.635 (0.405–0.997)	0.048
Antioxidants related factor	-0.297 (0.166)	3.2	0.743 (0.537–1.028)	0.073

B-Beta; SE-Standard error; CI-Confidence interval; OR-Odds ratio.

**Figure 1:**
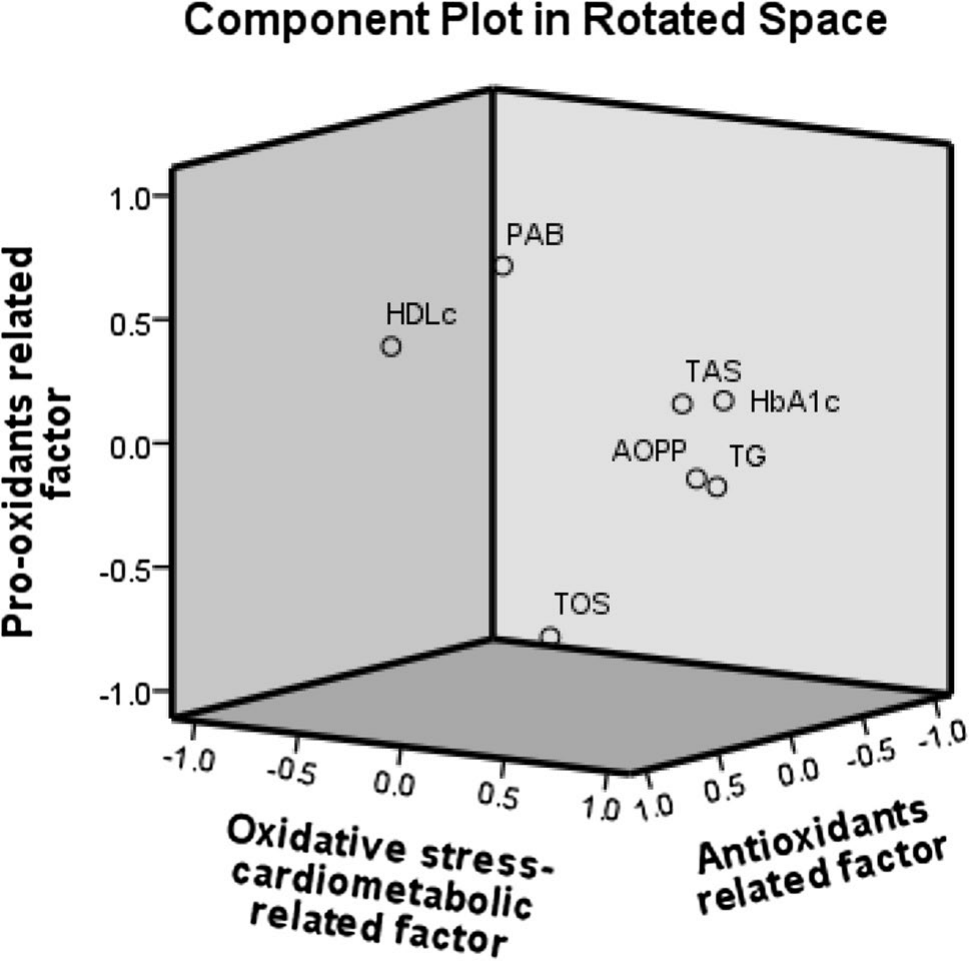
Principal component analysis: component plot in rotated space.

PCA results are accompanied by the graph, i.e. component plot in rotated space (Fig. 1).

## Discussion

To the best of our knowledge, this is the first study that investigated a mutual involvement of a cluster of redox balance homeostasis parameters, lipid parameters/indexes (i.e., CRI-I and CRI-II) and inflammation in patients with NAFLD.

Even though oxidative stress represents the underlying feature of NAFLD, there is no any single biomarker that could be regarded as a gold standard for redox status determination of this metabolic disorder so far^2^.

We have shown increased levels of pro-oxidants (i.e., AOPP, TOS and OSI) in subjects with NAFLD. However, the increased levels of antioxidants (i.e., TAS) observed in this study might be in part related to enhanced compensatory mechanism to increased production of ROS in NAFLD, given the fact that enhanced antioxidant defense system tries to cope with increased free radicals production.

Since controversial results were obtained by different studies regarding antioxidant levels/activities^4,14,15^, we have decided to measure the TAS levels which detects simultaneously all antioxidants without elimination of their mutual interactions^16^. Similarly, TOS reflects the measure of the overall pro-oxidants, whereas their ratio (i.e. TOS/TAS, so called OSI) represents the overall oxidative stress status. In addition, beside AOPP which reflects the measure of oxidative damage of proteins^9^, we have examined pro-oxidant-antioxidant balance (PAB) as a potentially better representative of simultaneous antioxidants and pro-oxidants in the same assay than each pro-oxidant determined separately. It was obtained by the ratio of uric acid and hydrogen peroxide and its increased serum values are indicative of higher production of ROS/RNS^10^.

Our results are in accordance with Başkol et al. who also reported higher AOPP, TOS and OSI in patients with NASH, although they included a smaller number of participants than our study did (i.e., a total of 28 patients with NASH and 19 healthy controls). Contrary to the results of the current study, they did not observe the difference in TAS levels between examined groups^17^.

In addition to oxidative stress, increased CRI-I, CRI-II and hsCRP in NAFLD that are demonstrated in this study are in line with our previous reports of involvement of dyslipidemia and inflammation in NAFLD^18–20^. The current findings are further confirmed by multivariate binary logistic regression analysis, thus extending our previous results, since we were limited earlier to confirm the diagnosis of NAFLD by abdominal ultrasound, but only with fatty liver index (FLI), and hepatic steatosis index (HIS), as proxy of NAFLD, both in diabetic and non-diabetic subjects^18–20^.

We have also shown higher HbA1c levels in NAFLD patients which might be explained by higher number of participants with T2DM in this group, compared to non-NAFLD counterparts. Previous cross-sectional and longitudinal studies have confirmed an independent relationship between HbA1c and NAFLD^21,22^. This might explain the tight connection between NAFLD, T2DM and obesity since insulin resistance mediated by oxidative stress and inflammation represent the common soil of these disorders^18,19,23^. In line with this, we have recently shown an independent association between HbA1c and comprehensive DOI score (i.e. dyslipidemia, oxidative stress and inflammation score) in patients with prediabetes and T2DM^24^.

Insulin resistance is regarded to be the hallmark of NAFLD, leading to increased FFA flux in the liver. This process originates from increased visceral adipose compartments that secrete a variety of proinflammatory adipokines and cytokines, as well as higher levels od FFA which compromise signalling pathways of insulin^25^. Furthermore, mitochondrial dysfunction characterized by increased ROS generation during the process of oxidative phosphorylation and liver fat peroxidation, represents the typical pathophysiological trait in NAFLD^5,25^. Since the insulin's anti-lipolytic effects are attenuated, enhanced lipolysis of TG, increased secretion of FFA and overwhelmed production of ROS lead to structural and functional hepatic changes^5,25^.

Another consequence of insulin resistance status is redistribution of HDL particles to smaller HDL3 ones which exhibit proatherogenic properties. Also, increased synthesis of sdLDL occurs^26^.

To further explore the interrelationship between oxidative stress, inflammation and metabolic disturbances, respectively and NAFLD, the PCA was applied. This analysis extracted 3 different factors explaining 68% of variance of the examined biomarkers. Oxidative stress-cardiometabolic related factor showed the highest percentage of variance (36%) with positive loadings of AOPP, TG, and HbA1c and with negative loading of HDL-c. The second factor explained 17% of the variance and consisted of Pro-oxidants related factor (i.e., TOS with negative loading and PAB with positive loading), whereas Antioxidants related factor explained 15% of the variance (i.e., TAS with negative loading). Furthermore, Oxidative stress-cardiometabolic related factor (i.e., TG, AOPP, HDL-c and HbA1c) and Pro-oxidants related factor (i.e., TOS and PAB) confirmed significant predictive ability towards NAFLD status, whereas Antioxidants related factor (i.e., TAS) lost its prediction. The obtained results further support the notion that oxidative stress-induced cardiometabolic disturbances could be potential risk factors for NAFLD.

Beside the cross-sectional design of the current research which limits us to conclude the causality between examined biomarkers and NAFLD, this is a single-center study which is another limitation since it does not allow us to generalize these results, given the fact that ethnicity and race may also influence the prevalence of NAFLD^3^. Thus, the obtained results can not be applied to non-Caucasian population.

Also, environmental factors such as nutritional habits and/or regular physical activity were not taken into account since both of them may lead to induction of antioxidative enzymes activity, as well as to increase in antioxidant molecules synthesis^27^.

Moreover, we were not able to diagnose NAFLD by magnetic resonance imaging or liver biopsy, which are more reliable diagnostic procedures than abdominal ultrasound.

Also, since this was a cross-sectional study, we could not provide information if these markers of oxidative stress fluctuate before and after the treatment. Future studies are needed to explore this issue.

Nevertheless, the strengths of our study should also be emphasized. Beside the relatively larger sample of participants included as compared to other studies^17^, we have examined a variety of parameters of redox homeostasis. Moreover, the current study is the first one that performed PCA to evaluate the mutual involvement of a cluster of redox balance homeostasis and cardiometabolic parameters to gain deeper knowledge into the pathological traits of NAFLD. Finally, lipid indexes, such as CRI-I and CRI-II can add significant contribution in discrimination between NAFLD and non-NAFLD patients.

## Conclusion

In addition to oxidative stress (i.e., determined by higher AOPP levels), dyslipidemia (i.e. determined by higher lipid indexes, CRI-I and CRI-II) and inflammation (determined by higher hsCRP) are tightly and independently related to NAFLD status. The mutual involvement of pro-oxidants (i.e. TOS and PAB), or the joint involvement of pro-oxidants (i.e. AOPP) and cardiometabolic parameters (i.e. HbA1c, TG and HDL-c) can differentiate subjects with NAFLD from those individuals without this metabolic disorder. Future studies are needed to confirm and extend our results in order to find the best therapeutic option for NAFLD treatment, since universal therapeutic target has not known yet.

## Data Availability

The data will be available upon reasonable request (contact person: aleksandranklisic@gmail.com).
